# Staging metastatic urothelial cancer with Nectin‐4 imaging using Gallium‐68‐N188 PET/CT

**DOI:** 10.1111/bju.16901

**Published:** 2025-08-23

**Authors:** Matthias Miederer, Marc Pretze, Elena Abbate, Andreas Hartig, Joana do Mar Ferreira Machado, Katharina Böhm, Ulrich Sommer, Sebastian Hoberück, Ralph A. Bundschuh, Jörg Kotzerke, Christian Thomas

**Affiliations:** ^1^ Department of Translational Imaging in Oncology, National Center for Tumor Diseases (NCT), NCT/UCC Dresden, a partnership between DKFZ, Faculty of Medicine and University Hospital Carl Gustav Carus TUD Dresden University of Technology, and Helmholtz‐Zentrum Dresden‐Rossendorf (HZDR) Dresden Germany; ^2^ Department of Nuclear Medicine, University Hospital Carl Gustav Carus Technische Universität Dresden Dresden Germany; ^3^ Department of Urology, University Hospital Carl Gustav Carus Technische Universität Dresden Dresden Germany; ^4^ Department of Pathology, University Hospital Carl Gustav Carus Technische Universität Dresden Dresden Germany

**Keywords:** Nectin‐4, ^68^Ga‐N188, positron emission tomography, PET/CT, enfortumab vedotin

## Abstract

**Objective:**

To quantify Nectin‐4 expression in tumour lesions using the Nectin‐4‐binding peptide Gallium‐68‐N188 and positron emission tomography (PET)/computed tomography (CT) in patients with advanced or metastasised urothelial cancer eligible for therapy with the Nectin‐4‐directed antibody–drug conjugate enfortumab vedotin, in combination with checkpoint inhibitor pembrolizumab (Ev/P).

**Methods:**

In 10 patients, Nectin‐4 PET/CT imaging was analysed before planned systemic therapy with Ev/P based on standardised uptake value (SUV) measurements and the results were correlated to available microscopic findings on Nectin‐4 immunohistochemistry and to clinical follow‐up.

**Results:**

Nectin‐4 PET is suitable for detecting Nectin‐4 expression in tumour lesions and demonstrates heterogeneity in Nectin‐4 expression – for example, between lymph node metastases and organ metastases. PET imaging of Nectin‐4 expression is therefore a potentially clinically relevant method for managing the application of Nectin‐4‐targeted therapies.

**Conclusions:**

We show, as a proof of principle, that Nectin‐4 expression can be detected on imaging and serves as an innovative biomarker for targeted therapy in urothelial cancer. Intra‐individual heterogeneous expression of Nectin‐4 in metastatic sites is frequent.

**Abbreviations**
ADC: antibody–drug conjugate
Ev/P: enfortumab vedotin plus pembrolizumab
^68^Ga: Gallium‐68
IHC: immunohistochemistry
SUV: standardised uptake value

## Introduction

Metastatic urothelial carcinoma is an aggressive disease associated with poor prognosis. However, several recent advances in therapy, such as immune checkpoint inhibitors and antibody–drug conjugates (ADCs), have revolutionised systemic treatment in this setting. Specifically, the ADC directed against Nectin‐4, enfortumab vedotin, has shown high efficacy [1]. Nectin‐4 is expressed in the majority of metastatic urothelial carcinomas of the bladder and is also found in non‐urothelial genitourinary malignancies [2]. It is an immunoglobulin‐like cell adhesion transmembrane protein involved in the development and maintenance of adherent junctions and is normally expressed in embryonic and placental tissues during fetal development. Because it is expressed in several other tumour entities, such as breast cancer, gastrointestinal tumours and lung cancer, Nectin‐4 is one of the most promising pan cancer targets [3, 4, 5, 6]. In advanced urothelial carcinoma, the EV‐302 trial showed that enfortumab vedotin plus pembrolizumab (Ev/P) was associated with an overall survival benefit compared to standard chemotherapy, the median overall survival being twice as long with Ev/P treatment [7]. Recently, several studies have reported that tissue‐based Nectin‐4 expression may serve as a surrogate marker for treatment response. However, these studies were limited by their retrospective nature [8, 9]. The role of Nectin‐4 targeting in successful treatment, therefore, has not been comprehensively elucidated, and it remains unclear whether this ADC combined with immune‐checkpoint inhibitors acts synergistically or whether bystander effects are likely to influence the relationship between targeting and the efficacy of enfortumab vedotin therapy.

Although more insight regarding the role of Nectin‐4 as a molecular target is still needed, Nectin‐4 expression is hypothesised to influence the efficacy of enfortumab vedotin to some extent and the clinical measurement of drug targets is a potential way to individualise therapy and guide future developments [10]. However, immunohistochemical Nectin‐4 quantification has several limitations. First, it is based on invasive tissue sampling. Second, it reflects the biology of single sites and might not reflect the current status of disease when archival samples are used. Third, heterogeneity at a microscopic level is difficult to interpret. These findings, combined with the evidence of a complex relationship between targeting and efficacy, might limit the validity of tissue‐based Nectin‐4 detection. Therefore, various PET imaging methods have been proposed, such as zirkonium‐89‐Nectin‐immuno‐PET or iodine‐124/iodine‐125‐enfortumab vedotin [11, 12]. PET imaging is a different method for measuring targeted expression that, to some extent, correlates with the results from immunohistochemistry (IHC). Nevertheless, the study of imaging targets for ADC therapy is a new field, with several aspects (such as resolution and quantification) that do not share properties with IHC. Most importantly, imaging can provide information on all measurable lesions in a patient at a given time and also provides a better reflection of the availability of the target for systemic treatment. Generally, imaging – in contrast to IHC – is a macroscopic method for measuring target expression that can only be compared to some extent to IHC, which reflects the microscopic situation. Thus, the development of PET probes is an important field that is informed by results from IHC but remains an independent method for visualisation and quantification of target expression.

As routine PET imaging typically utilises short‐lived isotopes, such as Gallium‐68 (^68^Ga) or Fluorine‐18 (^18^F), small‐molecule PET probes are desirable. For imaging of Nectin‐4, derivatives of bicyclic Nectin‐4‐binding peptides have been suggested [13]. Nectin‐4‐binding bicyclic peptides have been developed and described in clinical application to serve as small‐molecule counterparts to monoclonal antibodies for targeted drug conjugate therapy [14]. The ^68^Ga‐labelled Nectin‐4‐binding peptide N188 has been extensively evaluated preclinically and clinically, and shows promising properties in depicting Nectin‐4 [15, 16].

To guide therapeutic decisions in advanced and metastasised urothelial cancer, we perform ^68^Ga‐N188 PET/CT in patients who are eligible for Nectin‐4‐targeted therapy. Here, we report our single‐centre experience in our first 10 successive patients.

## Methods

### Patients

Gallium‐68‐N188 PET/CT was performed when recommended by an interdisciplinary tumour board to serve as an additional marker for Nectin‐4 tumour expression in patients with locally advanced/metastatic urothelial carcinoma planned for Ev/P treatment. Patients gave informed consent for data analysis and the competent ethics committee approved the retrospective study (BO‐EK‐438112024). Clinical follow‐up was analysed according to the hospital charts.

### Immunohistochemistry

#### Staining and Analysis

The immunohistochemical stains for Nectin‐4 were prepared from 4‐μm sections of all available formalin‐fixed paraffin‐embedded tumour samples, including the primary tumour and/or metastases. The immunohistochemical stains for Nectin‐4 (EPR15613‐68, Abcam Ltd; monoclonal, dilution 1:200) were prepared from 4‐μm sections of all available formalin‐fixed paraffin‐embedded tumour samples from each patient's cystectomy, including any lymph node metastases. The immunohistochemical staining was evaluated by an experienced pathologist, and designation of the respective H‐score was applied using the formula: percentage of weak nuclear and/or cytoplasmic staining + percentage of moderate staining × 2 + percentage of strong staining × 3 [17, 18].

#### ^68^Gallium‐N188 PET/CT

Gallium‐68 was eluted from an onsite ^68^Ge/^68^Ga generator (GalliaPharm, Eckert&Ziegler, Berlin, Germany) and the precursor N188 (*M* = 2546 g/mol) was kindly provided by Xing Yang and Xiaojiang Duan from Peking University Health Science Center, China (Data S2). PET/CT acquisition was performed 60–100 min after i.v. injection of 1.6 MBq/kg (±0.5 MBq) on a Biograph Vision 600 system (Siemens, Erlangen, Germany). PET images were reconstructed using an ordered subset expectation maximisation algorithm with four iterations and five subsets, applying point spread function, time of flight, attenuation and relative scatter correction.

#### Image Analysis

The available clinical imaging was reviewed and tumour lesions in the lung, bone, liver and lymphatic system were delineated using a semi‐automatic segmentation tool (IMAX EE, AGFA®HealthCare, N.V., Belgium) on contrast‐enhanced CT at the time of the PET/CT and during follow‐up after Ev/P therapy. Bone lesions present at baseline were delineated using MRI in one case. Standardised uptake values (SUVs) normalised to body weight as SUV_max_ (representing one voxel with highest SUV), SUV_peak_ (representing a clustered group of highest uptake) and SUV_mean_ were defined within the manually drawn volumes of interests informed by contrast‐enhanced CT (or MRI) with Syngo.Via VB80 (Siemens, Erlangen, Germany). Tumour response to Ev/P therapy was determined by manual delineation of index lesions in CT scans before and during treatment.

## Results

### Radiolabelling

Synthesis and quality control resulted in ^68^Ga‐N188 with radiochemical yields >90% and radiochemical purities >95%. The pH was in the range of 4.2–4.6 and endotoxin levels were always <5.0 EU/mL (Data S1). Different precursor amounts in the range 10–50 μg (4–20 nmol) resulted in different molar activities ranging from 3.6 to 216 MBq/nmol, with a mean amount of 13.8 nmol N188 labelled with 480 MBq ^68^Ga (Data S2).

### Patients

The study included data from 10 successive patients. Their mean age was 70 years at time of Nectin‐4 PET/CT. Primary and secondary metastatic urothelial cancer were present in four and five patients, respectively. In one patient, there was no clinical evidence of metastatic burden in any of the conducted imaging modalities but surgery revealed locally advanced cancer with positive resection margins. The mean length of follow‐up after diagnosis of metastatic disease was 342 days (interquartile range 147 days). Seven of 10 patients received radical surgery for the primary tumour, which was located in the bladder and in the upper urothelial tract in seven and three patients, respectively (Table 1). Eight of the 10 patients received systemic treatment with Ev/P after Nectin‐4 PET/CT. Two patients were not eligible for systemic treatment because they had poor performance status. Follow‐up imaging with contrast‐enhanced CT during systemic treatment with Ev/P was available for four patients with distant metastases. The mean time between morphological baseline imaging and follow‐up with conventional CT was 83 days (±21 days).

**Table 1:** Patient characteristics.

Patient	Gender	Age	Primary	Surgery	Metastatic stage at diagnosis	Previous systemic therapies	Enfortumab vedotin/pembrolizumab	Follow‐up after diagnosis of metastases, days	Death caused by cancer
1	M	65	Bladder	Yes	Secondary	Gem/Cis	Yes	229	Yes
2	M	67	Bladder	No	Primarily	ddMVAC	Yes	351	No
3	M	76	Bladder	No	Primarily	Gem/Cis, CPI	Yes	861	No
4	F	47	Bladder	Yes	Secondary	Gem/Cis	Yes	303	No
5	F	74	Bladder	Yes	None	–	Yes	−/−	No
6	M	76	Renal pelvis	No	Primarily	–	No	92	No
7	F	78	Renal pelvis	Yes	Secondary	CPI	No	567	Yes
8	M	85	Renal pelvis	Yes	Primarily	–	Yes	340	No
9	M	79	Bladder	Yes	Secondary	–	Yes	204	No
10	M	49	Bladder	Yes	Secondary	Gem/Cis	Yes	135	No

Cis, cisplatin; CPI, immune checkpoint inhibitor; ddMVAC, dose‐dense methotrexate, vinblastin, adriamycin, cispatin; Gem, gemcitabine.

### PET/CT Imaging

**Fig. 1:**
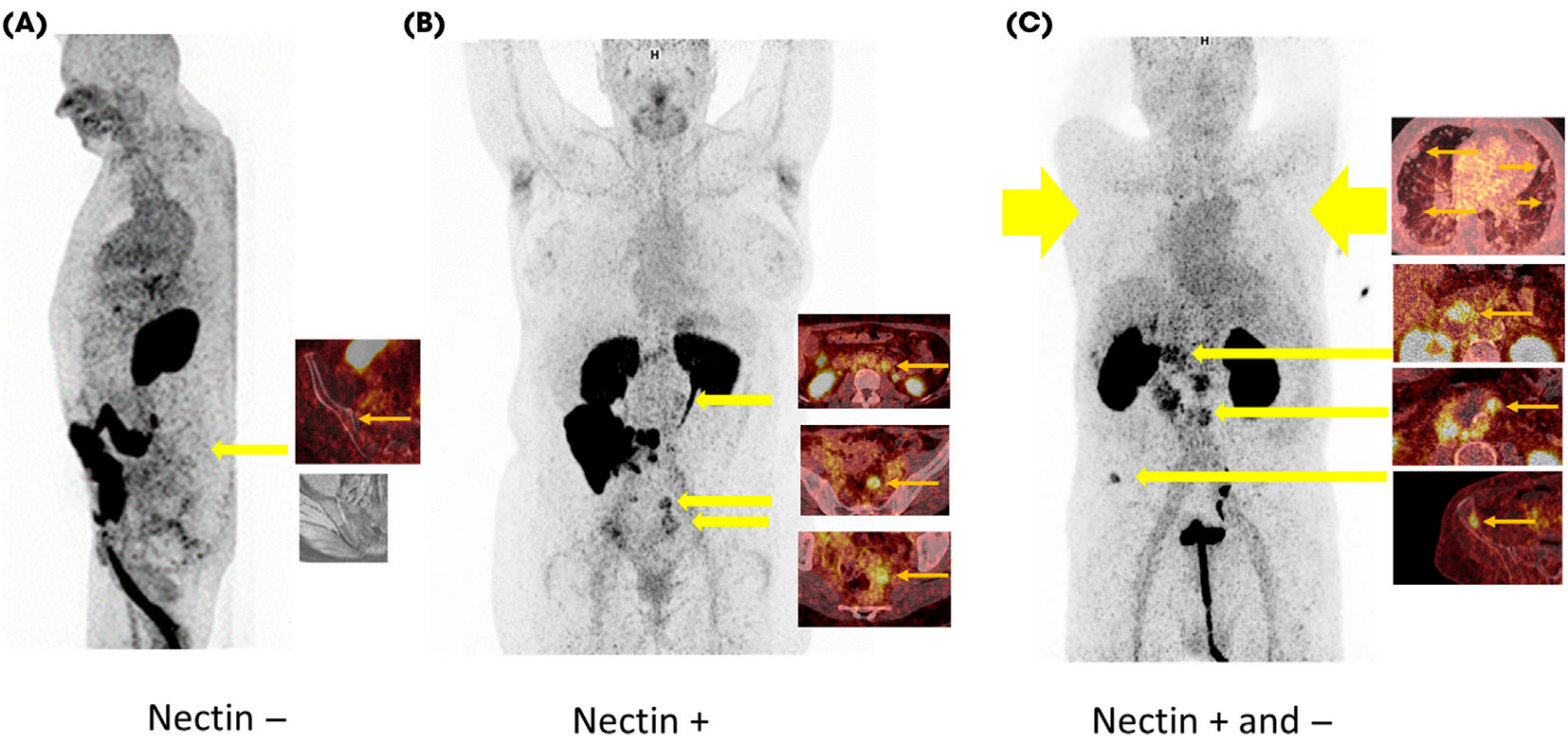
Example images depicting maximum intensity overviews and fusion images of representative metastases (standardised uptake value range 0–5). (**A**) Unremarkable Nectin‐4 PET/CT with known bone metastases, for example, at the right iliac bone. (**B**) Nectin‐4‐positive retroperitoneal lymph node metastases. (**C**) Nectin‐4‐positive lymph node metastases, one Nectin‐4‐positive bone metastasis, and images of Nectin‐4‐negative pulmonary metastases.

The PET/CT images showed ^68^Ga‐N188 uptake into tumour lesions at levels that were in the majority above liver background and within the range of blood pool background. However, there were some patients with visually negative metastases, demonstrating the expected heterogeneity in target expression (Fig. 1). Blood retention of the tracer is attributed to the pharmacokinetic properties of bicyclic peptides. To ensure good tumour to background ratios, imaging was started between 60 and 90 min (78 min, ±12 min) after injection. Although regarding optimal tumour accumulation of the tracer, earlier PET/CT time points might be possible, contrast between blood background and tumour might be lower at earlier time points, which might in particular improve detection of lymph node metastases adjacent to large vessels [16]. A The trend towards higher blood retention with higher amounts of tracer (that cannot be attributed to variations in distribution times) shows that the pharmacokinetics are dependent on the amount of tracer typical for tracers with long blood clearance (Fig. 3A).

### Nectin‐4 Expression Measurement by IHC and PET

**Fig. 2:**
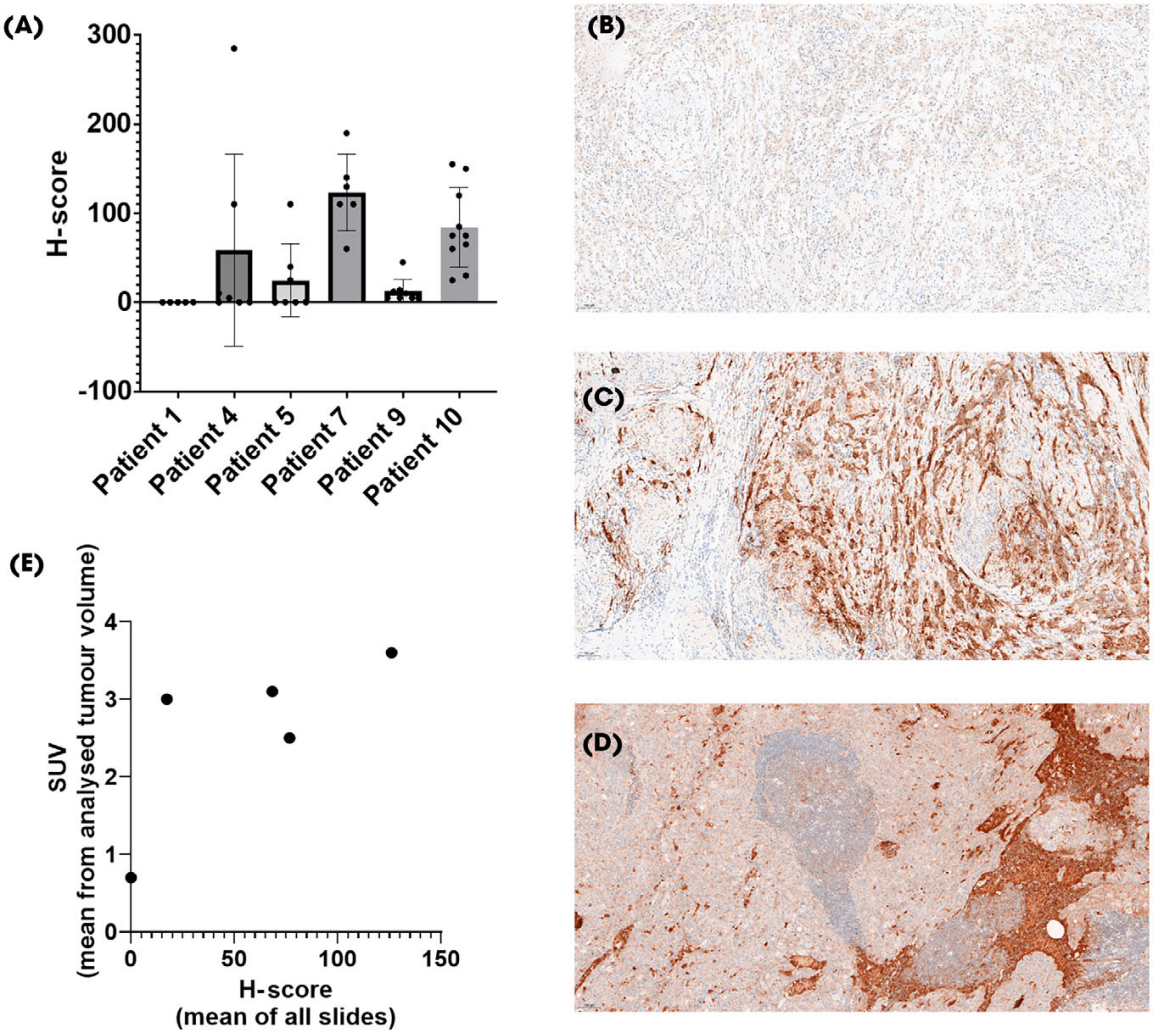
Nectin‐4 expression scored on immunohistochemistry (IHC) in all available slides. H‐score was calculated by adding weighted percentage of weak staining, moderate staining and strong staining, resulting in a score ranging from 0 to 300. (**A**) Bar chart with H‐scores from available tissue specimens in six patients. (**B–D**) Examples illustrating microscopic heterogeneity of Nectin‐4 expression. (**B**) Negative tumour parts. (**C**) Positive tumour parts. (**D**) Lymph node with positive and negative parts. (**E**) In all patients where both IHC scores and standardised uptake value (SUV) measurements were available for each patient, IHC scores were averaged over all slides and SUV_mean_ values were calculated from the sum of all tumour volumes analysed.

Tumour tissue for Nectin‐4 staining was available in six of the 10 patients. Relevant Nectin‐4 expression in tumour samples was observed in only two of the six patients. One patient had moderate to strong expression (H‐score 110) only in the lymph node metastasis, while expression in the primary tumour was low (H‐score 11). The other patient had moderate Nectin‐4 expression in the lymph node metastasis (H‐score 110) but markedly greater expression in the primary tumour (H‐score 285). The other four patients had only weak or no Nectin‐4 expression in most of the analysed tumour samples (mean H‐Score ≤ 10). For descriptive reasons, the means of all obtained H‐scores and SUVs were additionally correlated for individual patients, where both were available (Fig. 2).

**Fig. 3:**
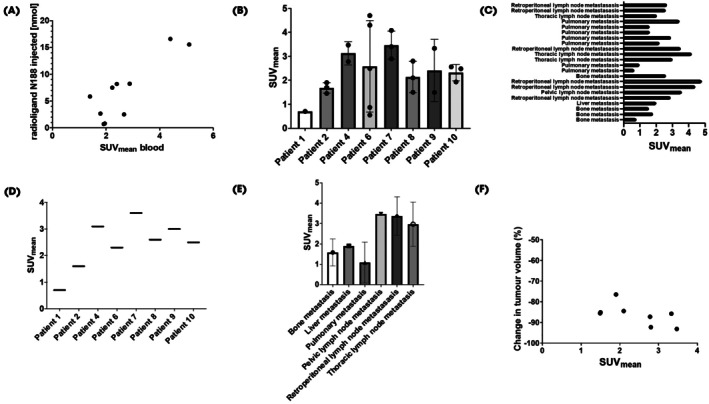
Nectin‐4 expression quantified using standardised uptake value (SUV) on Gallium‐68 (^68^Ga)‐N188 PET/CT. (**A**) Association between molar amount of used tracer (nmol) and blood pool background measured in the thoracic aorta. (**B**) Overview SUV_mean_ of analysed metastasis in all patients. (**C**) SUV_mean_ for all measured metastases. (**D**) SUV_mean_ in all patients calculated as a volume weighted mean of SUV_mean_ for every patient (resulting in one SUV_mean_ over all measured metastases). (**E**) SUV_mean_ in metastases in all organ systems (measured SUV_mean_ showed in C). (**F**) Association of Nectin‐4 expression quantified with SUV_mean_ on ^68^Ga‐N188 PET/CT and change of tumour volume in lesions that could be delineated in CT before and during enfortumab vedotin plus pembrolizumab therapy. In these patients, the strong effect of therapy was clearly evident, but a clear association with SUV cannot be concluded.

Similarly to the heterogeneous expression at the microscopic level, we observed a heterogeneous tracer uptake of ^68^Ga‐N188 at different metastatic sites (Fig. 3C,E). Nectin‐4 was detectable by molecular imaging in lymph node metastasis, bone metastasis and lung metastasis in the majority of patients. There was a trend towards higher SUV_mean_ levels in lymph node metastases compared to visceral or bone metastases (Fig. 3E). As shown in Fig. 3B,D, only one patient was found to have visually complete negative bone metastasis and one patient showed both clearly negative and positive metastasis. One patient presented with liver metastasis which was visually not detectable on PET despite ^68^Ga‐N188 uptake in the metastatic lesion; this was attributed to high liver background. To ensure the reliability of SUV measurements, we assessed correlations between SUV_max_, SUV_peak_ and SUV_mean_, and observed high correlation among all three variables (Data S3).

### Clinical Follow‐Up and Response in Relation to PET

Conventional follow‐up imaging was available for four patients receiving Ev/P treatment, and the tumour volumes from eight index metastases were compared before and during therapy. Here, a strong effect of Ev/P was seen in all patients. All patients presented a partial response in the index lesions. There was a trend towards a greater change in tumour volume with increasing SUV_mean_ levels and for increasing ratio of the tumour SUV_mean_ to blood background SUV (Fig. 3F and Data S4). Six of eight patients are still alive after a mean follow‐up of 347 days (Data S5).

## Discussion

Monoclonal antibodies are a class of molecules that are highly suited to target a variety of diseases and thus serve not only as a basis for improving diagnostics but also for development of new therapeutic regimens. For example, by blocking the PD1–PDL1 interaction, the immune system can be tailored strongly towards antitumour activity. The specific binding of monoclonal antibodies has also been increasingly utilised to carry cytotoxic substances towards tumour tissue and thereby reduce systemic toxicity over untargeted drugs. These ADCs are a new and highly promising class of systemic treatment. The primary antitumour action of ADCs occurs through the targeting of tumour cells and the delivery of a cytotoxic payload to the tumour tissue. Ev/P significantly improved patient median overall survival (31.5 vs 16.3 months; hazard ratio 0.47) and median progression‐free survival (12.5 vs 6.3 months; hazard ratio 0.45), with acceptable treatment‐related adverse events, and has recently become the new standard in first‐line therapy for locally advanced and metastatic urothelial cancer [7, 19]. In the small cohort described here we confirmed the potential effect of Ev/P on urothelial tumours independent of metastatic site. A trend towards better efficacy with higher Nectin‐4 expression measured by PET might be concluded from our preliminary results. However, these data should be interpreted with caution, given the fact that we observed frequent heterogeneous expression at a microscopic and a macroscopic level. The most commonly used method to measure Nectin‐4 tumour expression is to stain tissue samples by IHC using validated monoclonal antibodies. Klumper et al. have shown by IHC and molecular analysis that, in patients receiving enfortumab monotherapy, high Nectin‐4 expression levels in tumour tissue correlate with improved survival [8, 9]. However, tumour tissue reflects only the biology of a single site and might not reveal the current status of the disease when archival samples are used. Data from Duan et al. [16] suggest that there is a strong correlation between Nectin‐4 expression in tumour tissue and in PET imaging. In our series, there was high inter‐ and intra‐individual Nectin‐4 expression at the microscopic and macroscopic level but the expected relationship between quantification of Nectin‐4 expression by IHC and by PET could also be seen. This is in line with reported PET uptake values for Nectin‐4 tracers being lower than for theranostic tracers that are already highly successful, such as PSMA ligands or somatostatin analogues. The excellent efficacy of Ev/P observed in our cohort despite heterogeneity in Nectin‐4 expression might also be explained by the fact that cell death of Nectin‐4 cells and its immediate cellular neighbourhood addressed by bystander effects stimulates the immune system and therefore efficacy of PD1 inhibition.

In summary, as a proof of principle, we show that Nectin‐4 expression in urothelial cancer can be quantified by ^68^Ga‐N188 PET/CT and has a potential role in individualising therapy in selected patients. For patient‐individualised quantification of Nectin‐4 expression, molecular imaging might be superior to analysis of tissue specimen. Inter‐ and intra‐individual heterogeneity of Nectin‐4 expression at a macroscopic level should be taken into account.

## Disclosure of Interests

MM reports consultant fees from Novartis, Roche, Telix, and Veraxa. EA received speakers' honoraria from Merck Sharp and Dohme, Astellas Pharma, Orion Pharma, Johnsons and Johnson Innovative Medicine and sponsoring by IPSEN Pharma and Merckgroup. KB reports payment or honoraria for lectures, presentations, speakers bureaus from Astellas and BMS. RAB is Consultant for and has received speaker's honoraria from Bayer Healthcare, Novartis, Terumo GmbH, and Eisai GmbH and has received travel expenses from Blue Earth Therapeutics. CM reports consulting fees from Astellas, Janssen, Bayer and MSD; payment or honoraria for lectures, presentations, speakers bureaus from Astellas, Janssen, Bayer and MSD; support for attending meetings and/or travel from Janssen and Ipsen; and serves as a board member of the German Society of Urology. For the other authors no conflicts exist.

## Supporting information


**Data S1.** (A) Representative chromatogram of 5 μg N188 precursor at *t*_R_ = 4.4 min (DMSO at *t*_R_ = 1.41 min). (B) Representative radiochromatogram of ^68^Ga‐N188 at *t*_R_ = 6.4 min (free ^68^Ga at *t*_R_ = 1.40 min) indicating >95% radiochemical purity.
**Data S2**. Radiolabeling Method and labeling yield: Radiosynthesis was performed automatically on a Modular‐Lab EAZY synthesis module (Eckert&Ziegler, Berlin, Germany) equipped with an EluGen syringe pump (Eckert&Ziegler, Berlin, Germany) for automatic generator elution. In brief, one or two generators were eluted with 5 mL 0.1 m HCl each onto an SCX cartridge (Eichrom, Lisle, IL, USA) for trapping the ^68^Ga. 10–50 μg (4–20 nmol) of precursor N188 in 50 μL DMSO was added to 300 μL sodium acetate buffer (1.1 m, pH 4.4–4.5) and 2 mg ascorbic acid. This solution was reacted for 8 minutes at 95°C with 0.8–1.2 GBq 68Ga eluate (800 μL 4.9 m NaCl 99.9999%/0.4% HClsuprapure) derived from the SCX cartridge. The reaction solution was purified by a CM light cartridge (WAT023531, Waters, Milford, MA, USA) and directly poured over a MILLEX‐GS 0.22 μm sterile filter (SLGVV255F) into the product vial. Additional 6 mL ringer acetate solution was added. Quality control was performed by thin‐layer chromatography on iTLC‐SG strips (Agilent, Santa Clara, CA, USA) for free 68Ga3+ (0.1 m citrate buffer pH 5) and for colloidal 68Ga species (1 m ammonium acetate:methanol 1:1). Additionally, radio‐HPLC was performed for determination of radiochemical purity and identity of 68Ga‐N188. The pH was measured using a reflectance photometer (QUANTOFIX Relax, Macherey‐Nagel GmbH & Co. KG, Düren, Germany). For endotoxin level determination, 10 μL of the product solution was diluted with 990 μL sterile water (1:100) using an EndoSafe PTS (Charles River, Sulzfeld, Germany).
**Data S3**. Different methods to measure SUV were correlated against each other showing the expected high concordance. (A) SUVmax and SUVpeak. (B) SUVmean and SUVpeak. (C) SUVmean and SUVmax.
**Data S4**. Association of Nectin‐4 expression quantified by the ratio of SUVmean in tumour to SUVmean of blood background and the change of tumour volume in lesions that could be delineated in CT before and during Ev/P therapy.
**Data S5**. Kaplan Meier analysis on overall survival in all patients having received a ^68^Ga‐N188 PET/CT.
