# Supplementary material for: Staging metastatic urothelial cancer with Nectin‐4 imaging using Gallium‐68‐N188 PET/CT
Source: BJU Int. 2025 Aug 23;137(1):166–72. doi: 10.1111/bju.16901 (PMC12690338; doi:10.1111/bju.16901)
Supplement: Supplementary file 1 — Data S1. (A) Representative chromatogram of 5 μg N188 precursor at t R = 4.4 min (DMSO at t R = 1.41 min). (B) Representative radiochromatogram of 68Ga‐N188 at t R = 6.4 min (free 68Ga at t R = 1.40 min) indicating >95% radiochemical purity. Data S2. Radiolabeling Method and labeling yield: Radiosynthesis was performed automatically on a Modular‐Lab EAZY synthesis module (Eckert&Ziegler, Berlin, Germany) equipped with an EluGen syringe pump (Eckert&Ziegler, Berlin, Germany) for automatic generator elution. In brief, one or two generators were eluted with 5 mL 0.1 m HCl each onto an SCX cartridge (Eichrom, Lisle, IL, USA) for trapping the 68Ga. 10–50 μg (4–20 nmol) of precursor N188 in 50 μL DMSO was added to 300 μL sodium acetate buffer (1.1 m, pH 4.4–4.5) and 2 mg ascorbic acid. This solution was reacted for 8 minutes at 95°C with 0.8–1.2 GBq 68Ga eluate (800 μL 4.9 m NaCl 99.9999%/0.4% HClsuprapure) derived from the SCX cartridge. The reaction solution was purified by a CM light cartridge (WAT023531, Waters, Milford, MA, USA) and directly poured over a MILLEX‐GS 0.22 μm sterile filter (SLGVV255F) into the product vial. Additional 6 mL ringer acetate solution was added. Quality control was performed by thin‐layer chromatography on iTLC‐SG strips (Agilent, Santa Clara, CA, USA) for free 68Ga3+ (0.1 m citrate buffer pH 5) and for colloidal 68Ga species (1 m ammonium acetate:methanol 1:1). Additionally, radio‐HPLC was performed for determination of radiochemical purity and identity of 68Ga‐N188. The pH was measured using a reflectance photometer (QUANTOFIX Relax, Macherey‐Nagel GmbH & Co. KG, Düren, Germany). For endotoxin level determination, 10 μL of the product solution was diluted with 990 μL sterile water (1:100) using an EndoSafe PTS (Charles River, Sulzfeld, Germany). Data S3. Different methods to measure SUV were correlated against each other showing the expected high concordance. (A) SUVmax and SUVpeak. (B) SUVmean and SUVpeak. (C) SUVmean and SUVmax. Data S4 [file BJU-137-166-s001.pptx]

## Slide 1
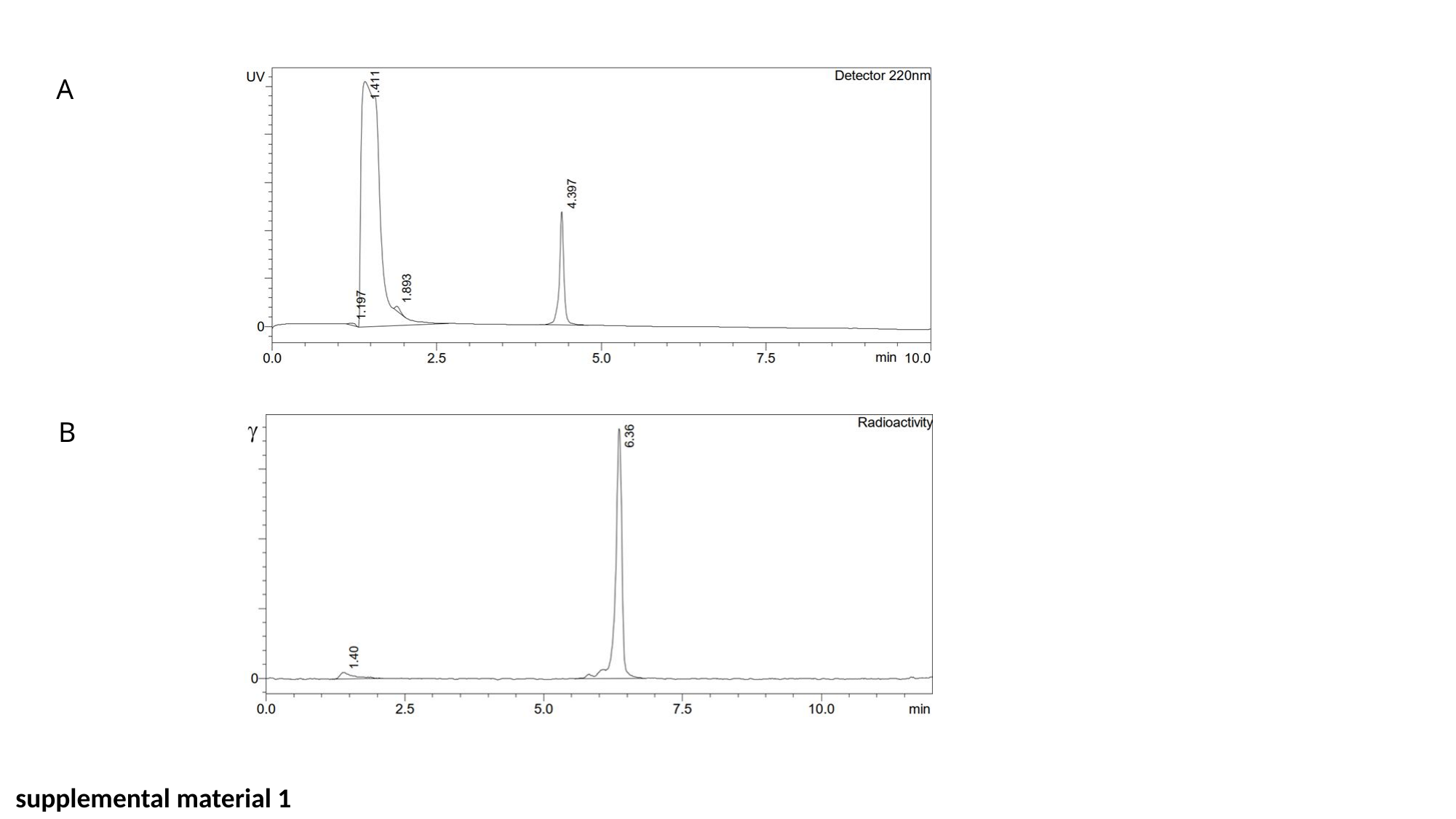

A
B
supplemental material 1

## Slide 2
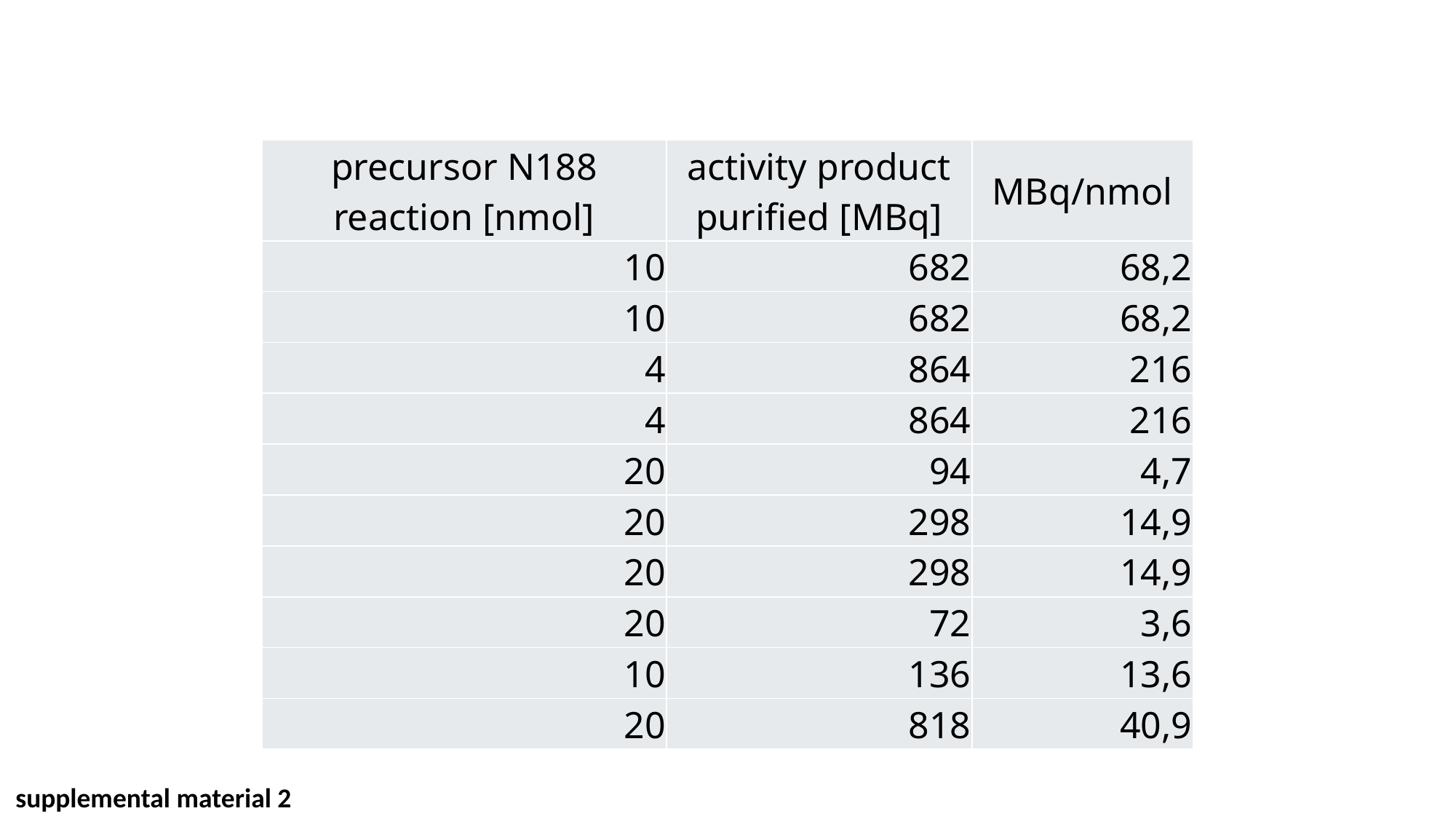

| precursor N188 reaction [nmol] | activity product purified [MBq] | MBq/nmol |
| --- | --- | --- |
| 10 | 682 | 68,2 |
| 10 | 682 | 68,2 |
| 4 | 864 | 216 |
| 4 | 864 | 216 |
| 20 | 94 | 4,7 |
| 20 | 298 | 14,9 |
| 20 | 298 | 14,9 |
| 20 | 72 | 3,6 |
| 10 | 136 | 13,6 |
| 20 | 818 | 40,9 |
supplemental material 2

## Slide 3
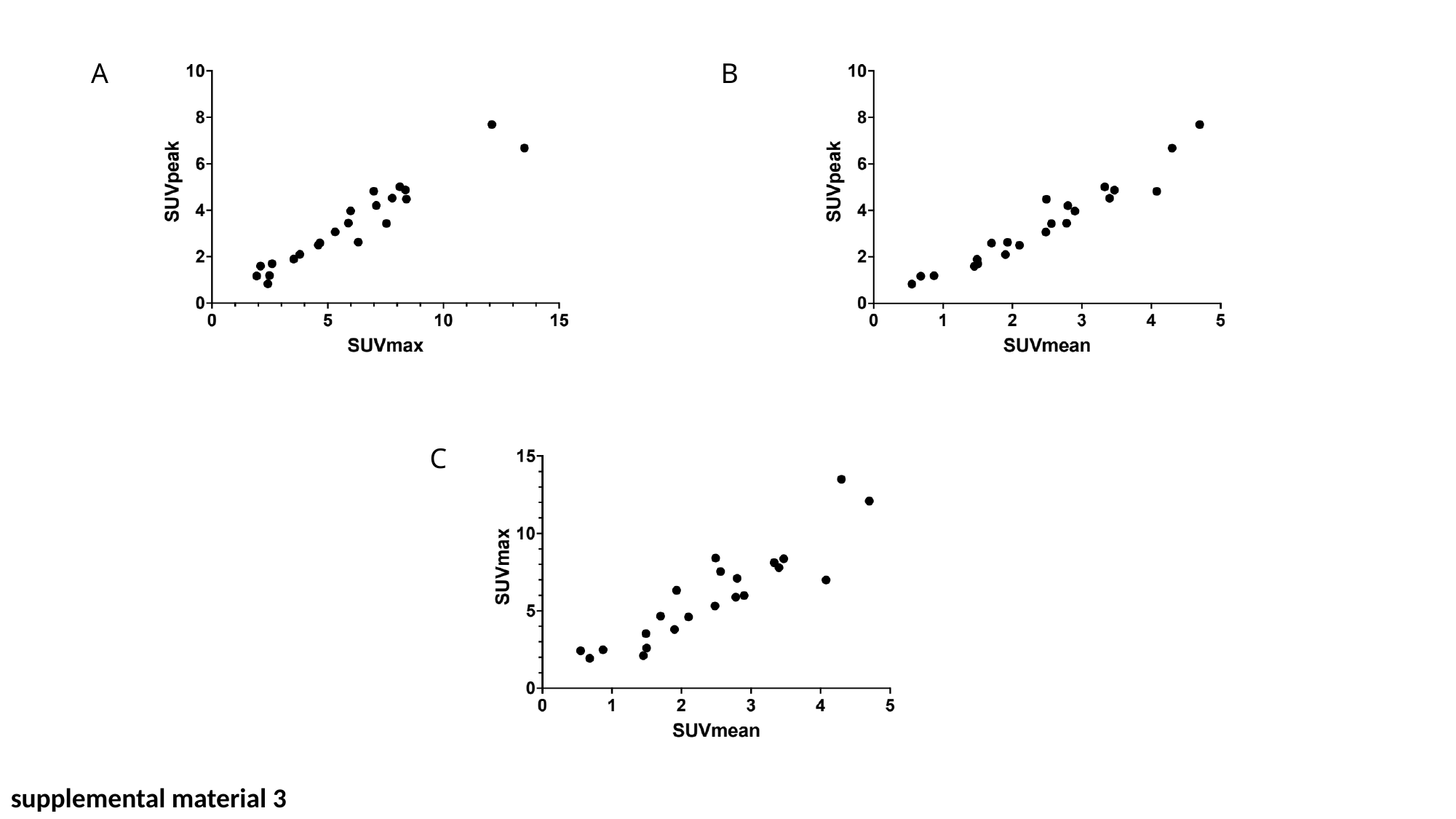

A
B
C
supplemental material 3

## Slide 4
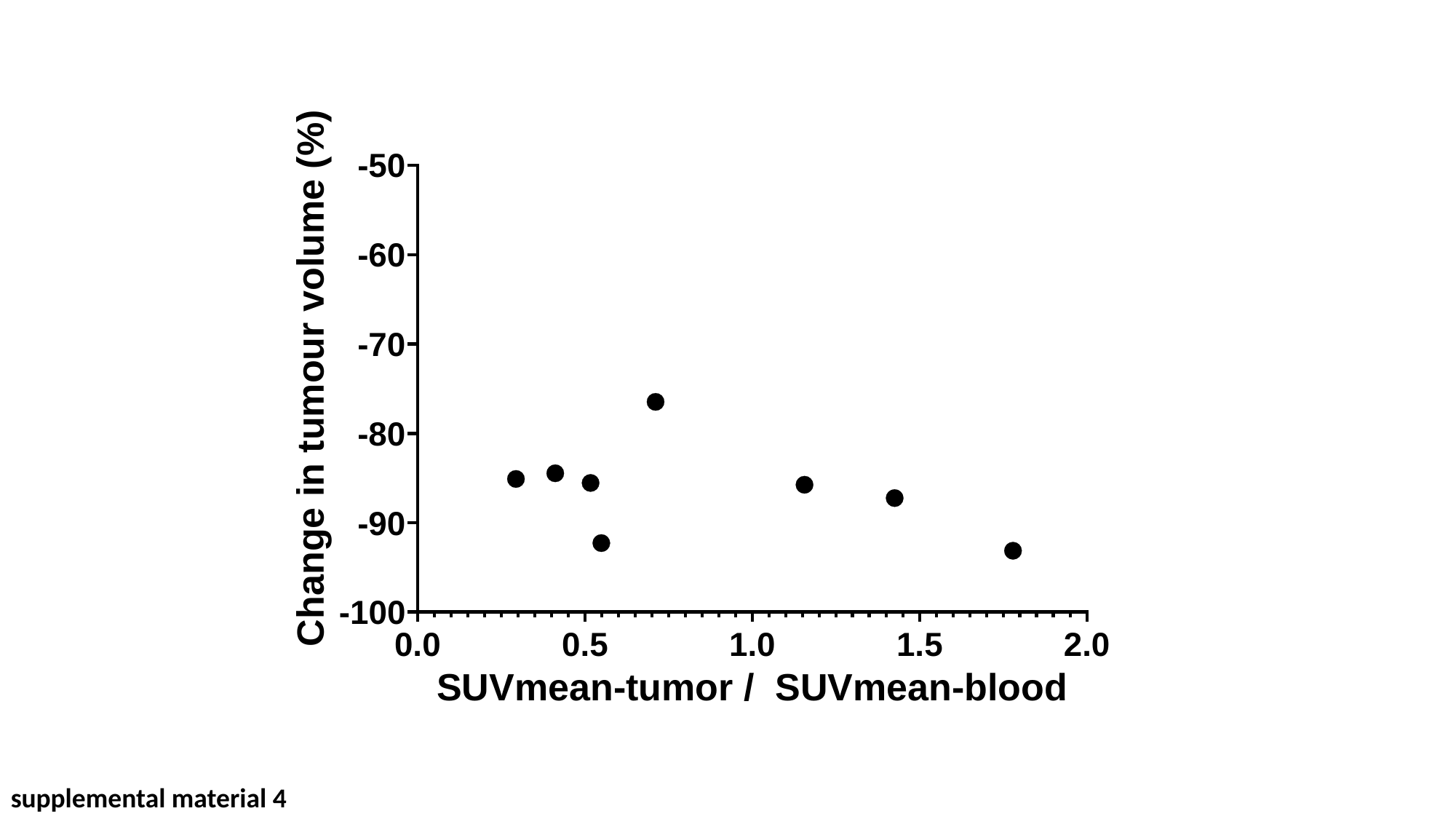

supplemental material 4

## Slide 5
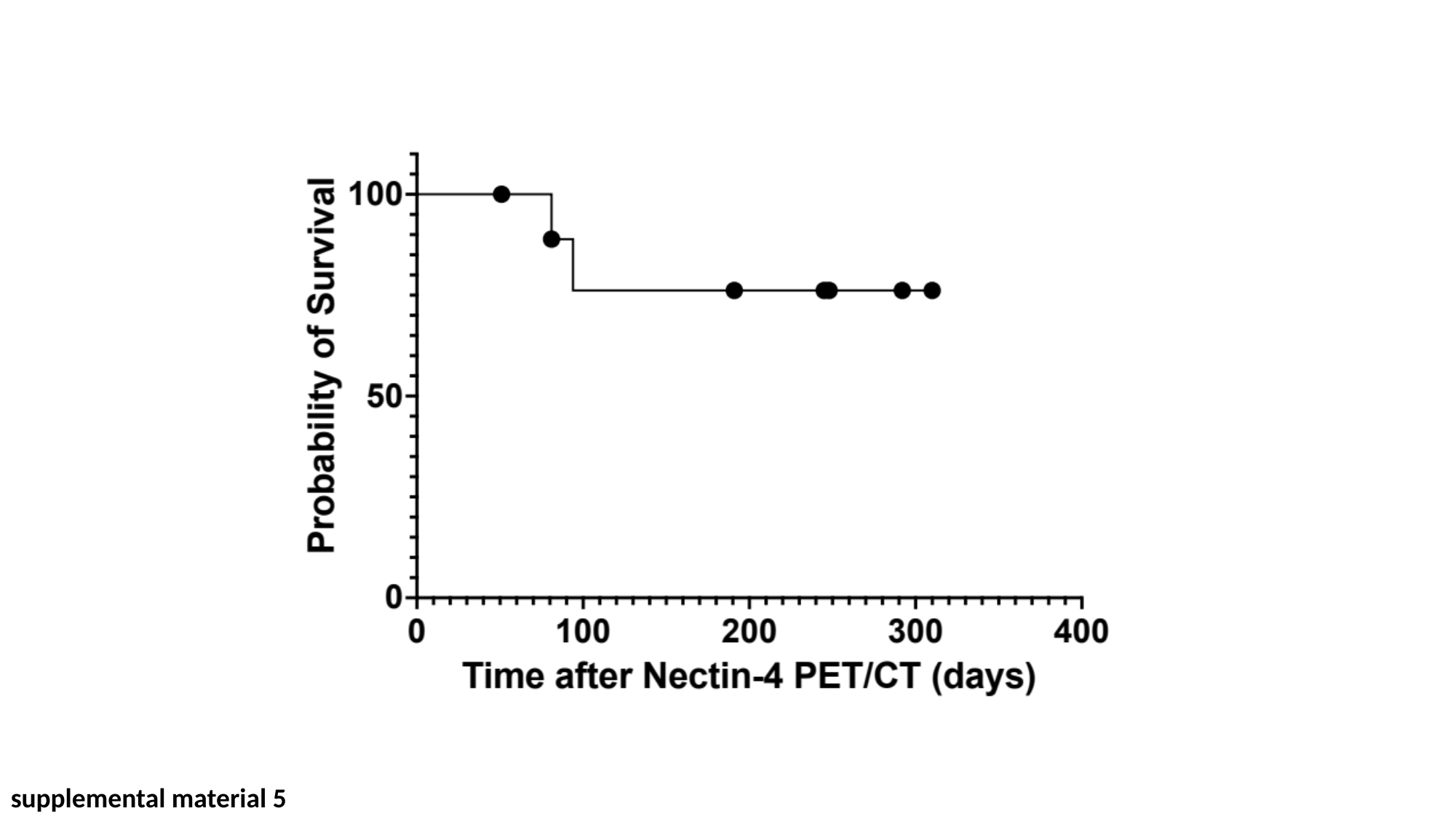

supplemental material 5
